# N-Acetylneuraminic Acid Supplementation Prevents High Fat Diet-Induced Insulin Resistance in Rats through Transcriptional and Nontranscriptional Mechanisms

**DOI:** 10.1155/2015/602313

**Published:** 2015-11-25

**Authors:** Zhang Yida, Mustapha Umar Imam, Maznah Ismail, Norsharina Ismail, Nur Hanisah Azmi, Waiteng Wong, Hadiza Altine Adamu, Nur Diyana Md Zamri, Aini Ideris, Maizaton Atmadini Abdullah

**Affiliations:** ^1^Laboratory of Molecular Biomedicine, Institute of Bioscience, Universiti Putra Malaysia, 43400 Serdang, Selangor, Malaysia; ^2^Cardiology Department, Affiliated Hospital of Chengde Medical University, Chengde, Hebei 067000, China; ^3^Department of Nutrition and Dietetics, Faculty of Medicine and Health Sciences, Universiti Putra Malaysia, 43400 Serdang, Selangor, Malaysia; ^4^Faculty of Veterinary Medicine, Universiti Putra Malaysia, 43400 Serdang, Selangor, Malaysia; ^5^Department of Pathology, Faculty of Medicine and Health Sciences, Universiti Putra Malaysia, 43400 Serdang, Selangor, Malaysia

## Abstract

N-Acetylneuraminic acid (Neu5Ac) is a biomarker of cardiometabolic diseases. In the present study, we tested the hypothesis that dietary Neu5Ac may improve cardiometabolic indices. A high fat diet (HFD) + Neu5Ac (50 or 400 mg/kg BW/day) was fed to rats and compared with HFD + simvastatin (10 mg/kg BW/day) or HFD alone for 12 weeks. Weights and serum biochemicals (lipid profile, oral glucose tolerance test, leptin, adiponectin, and insulin) were measured, and mRNA levels of insulin signaling genes were determined. The results indicated that low and high doses of sialic acid (SA) improved metabolic indices, although only the oral glucose tolerance test, serum triglycerides, leptin, and adiponectin were significantly better than those in the HFD and HFD + simvastatin groups (*P* < 0.05). Furthermore, the results showed that only high-dose SA significantly affected the transcription of hepatic and adipose tissue insulin signaling genes. The data suggested that SA prevented HFD-induced insulin resistance in rats after 12 weeks of administration through nontranscriptionally mediated biochemical changes that may have differentially sialylated glycoprotein structures at a low dose. At higher doses, SA induced transcriptional regulation of insulin signaling genes. These effects suggest that low and high doses of SA may produce similar metabolic outcomes in relation to insulin sensitivity through multiple mechanisms. These findings are worth studying further.

## 1. Introduction

Sialic acids (SAs) are* N*-acetylated derivatives of neuraminic acid that occur naturally in glycoproteins and gangliosides. SA is a biomarker of cardiometabolic diseases, where it is thought to be a consequence of long-term inflammation [1]. This claim is supported by the hypothesis that elevated SA levels in cardiovascular diseases may facilitate the resialylation of vascular endothelium in an attempt to reverse atherosclerosis [2]. Additionally, results of dietary supplementation with SA have not been consistent; some reports show that it promotes inflammation, hepatocellular cancer, and hemolytic-uremic syndrome [3, 4], while others have shown that it may be useful for brain development and for certain age-related disorders that cause reduced salivation [5, 6]. What can be gleaned from the reports thus far is that the nonhuman SA, N-glycolylneuraminic acid (Neu5Gc), not N-acetylneuraminic acid (Neu5Ac), is responsible for the deleterious effects of SA [4, 5].

There are numerous pharmacological and alternative therapies for cardiovascular diseases, but most of them have proven ineffective in curbing the rising burden of the diseases. This is driving the search for alternatives to currently available therapies [7]. Moreover, the rising burden of obesity and other risk factors for cardiometabolic diseases continually increase the prevalence of these diseases. If cardiometabolic diseases are to be effectively managed, there is a need for alternatives to the currently available therapies that control the risk factors and prevent the development of these diseases. To evaluate a potential alternative for the prevention of cardiometabolic diseases, we studied the effects of dietary supplementation with SA on the development of insulin resistance, which is a common denominator for cardiometabolic diseases [8]. Simvastatin is typically used to lower lipids, and because the animal model in the present study was given a diet rich in fats, it was used as a control. Additionally, we decided to use Neu5Ac since, which unlike Neu5Gc, it is the form that is widely reported to be beneficial. As a marker of cardiometabolic diseases, we hypothesized that dietary Neu5Ac could have far-reaching effects on cardiometabolic indices in view of the widespread incorporation of SA into multiple tissues in the body.

## 2. Materials and Methods

### 2.1. Materials

Neu5Ac was purchased from Carbosynth Limited (Compton, Berkshire, UK), while analytical grade ethanol was purchased from Merck (Darmstadt, Germany). Lipid profile kits were purchased from Randox Laboratories Ltd. (Crumlin, County Antrim, UK), while ELISA kits (leptin, insulin, and adiponectin) were purchased from Elabscience Biotechnology Co., Ltd. (Wuhan, China), and Millipore (Billerica, MA, USA), respectively. An RNA extraction kit was purchased from RBC Bioscience Corp. (Taipei, Taiwan), and a GenomeLab GeXP Start Kit was purchased from Beckman Coulter, Inc. (Miami, FL, USA). Simvastatin was purchased from Pfizer (New York, NY, USA), and RCL-2 solution was purchased from Alphelys (Toulouse, France). Cholesterol and cholic acid were purchased from Amresco (Solon, OH, USA) and Santa Cruz Biotechnology (Santa Cruz, CA, USA), respectively. Standard rat pellet was purchased from Specialty Feeds (Glen Forrest, WA, USA), while palm oil was supplied by Yee Lee Edible Oils Sdn. Bhd. (Perak, Malaysia).

### 2.2. Animal Study

The Animal Care and Use Committee (ACUC) of the Faculty of Medicine and Health Sciences, Universiti Putra Malaysia, approved the use of animals in this study (project approval number UPM/IACUC/AUP-R011/2014), and the animals were handled as stipulated by the guidelines for the use of animals. Sprague Dawley rats (10-week-old, 230–280 g, *n* = 30) were housed in individual cages at 25 ± 2°C, with 12/12 h light/dark cycle, and allowed to acclimatize for 2 weeks with free access to normal pellet and water. The rats were then assigned to one of five groups (*n* = 6, Table 1): normal group fed with standard rat pellet (335 Kcal/100 g), high fat diet (HFD) group fed a HFD alone (448 Kcal/100 g), simvastatin group fed with HFD + oral gavage of 10 mg/kg BW simvastatin/day (HFD + SIM), and SA groups that received HFD + daily oral gavage of 50 or 400 mg/kg BW SA (HFD + SAL and HFD + SAH, resp.). Simvastatin was chosen because it is the standard drug used to manage hyperlipidemia and associated metabolic perturbations [9], similar to what HFD induces in rats. Diets were formulated in-house except for the normal pellet and were given to the rats for 12 weeks, after which they were euthanized and their blood and organs (liver and visceral adipose tissues) were collected for further studies. Tissue samples were immediately washed with normal saline and preserved in chilled RCL-2 solution, which was then transferred to −80°C until RNA was extracted. During the intervention, food intake was calculated daily by subtracting the leftover food from what was added the previous day, while body weight was recorded.

### 2.3. Serum Adiponectin, Leptin, and Insulin

Serum from blood collected in plain tubes was used for measurements of adiponectin, leptin, and insulin using the respective ELISA kits according to the manufacturer's instructions. Absorbances were read on a BioTeK Synergy H1 Hybrid Reader (BioTek Instruments, Inc., Winooski, VT, USA) at the appropriate wavelengths (450 nm for insulin and leptin, and 450 nm and 590 nm for adiponectin). The results were analyzed on http://www.myassays.com/ using four parametric test curves: adiponectin (*R* ^2^ = 0.9914), insulin (*R* ^2^ = 1), and leptin (*R* ^2^ = 0.9996).

### 2.4. Biochemical Analyses

Lipid profile analyses were performed using serum from blood collected at the end of the study by cardiac puncture after an overnight fast. Samples were analyzed using Randox analytical kits according to the manufacturer's instructions with a Selectra XL instrument (Vita Scientific, Dieren, Netherlands). After an overnight fast, the oral glucose tolerance test (OGTT) was performed by oral gavage of 2 g/kg BW D-glucose to every rat, and the blood glucose levels were measured at 0, 30, 60, and 120 minutes via tail vein puncture, using a glucometer (Roche Diagnostics, Indianapolis, IN, USA). The area under the curve for glucose in the OGTT was calculated as reported previously [10]. Then, homeostatic model assessment of insulin resistance (HOMA-IR), a measure of insulin sensitivity, was computed from the fasting plasma glucose and insulin levels using the formula HOMA-IR = (fasting glucose level (mg/dL)/fasting plasma insulin (*μ*U/mL))/2430 [11].

### 2.5. Gene Expression Study

Primers for the gene expression study were designed using the* Rattus norvegicus* gene sequences from the National Center for Biotechnology Information website (http://www.ncbi.nlm.nih.gov/nucleotide/) and tagged with an 18-nucleotide universal forward and 19-nucleotide universal reverse sequence, respectively. Primers (Table 2) were supplied by Integrated DNA Technologies (Singapore) and reconstituted in RNAse free water. Extracted RNA (20 ng) from liver and adipose tissues was used for reverse transcription and PCR according to the GenomeLab GeXP Start Kit protocol (Beckman Coulter, USA) using the conditions shown in Table 2. PCR products (1 *μ*L) were analyzed on a GeXP genomelab genetic analysis system (Beckman Coulter, Inc., Miami, FL, USA) after mixing with sample loading solution and DNA size standard 400 as recommended by the manufacturer. Results were analyzed with the Fragment Analysis module of the GeXP system software and normalized on the eXpress Profiler software. Fold changes were calculated by dividing the expression value for the different treatment groups by the expression value for the normal group.

### 2.6. Data Analysis

The means ± standard deviation (*n* = 6) of the groups was used for the analyses. One-way analysis of variance (ANOVA) with Tukey's* post hoc* test was performed using SPSS 17.0 software (SPSS, Inc., Chicago, IL, USA) to assess the level of significance of the differences between means with a cutoff of *P* < 0.05.

## 3. Results

### 3.1. Body Weight Changes

**Figure 1:**
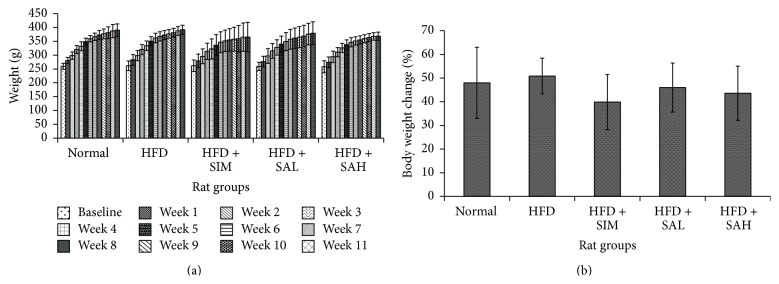
Effects of SA on body weight changes in HFD-fed rats over 12 weeks. (a) Total body weight changes. (b) Percentage of body weight changes. Groups are the same as Table 1. No significant differences were observed between the groups' actual weights, but total weight gain was the highest in the HFD and normal groups (50% and 47%, resp.), while the HFD + simvastatin group had the lowest (40% increase), followed by the HFD + SAH and HFD + SAL groups (42% and 46%, resp.).

Food intake was similar for all the groups throughout the intervention period (Table 3), but differences in weight gain were observed between the HFD and other groups at the end of 12 weeks (Figure 1); the highest weight gain between the beginning and end of the study (50%) was in the HFD group, followed by the normal group (47%), while the HFD + SIM group had the lowest weight gain (40%). The HFD + SAH and HFD + SAL groups had weight gains of 42% and 46%, respectively.

### 3.2. OGTT, Insulin, HOMA-IR, and Lipid Profile

**Figure 2:**
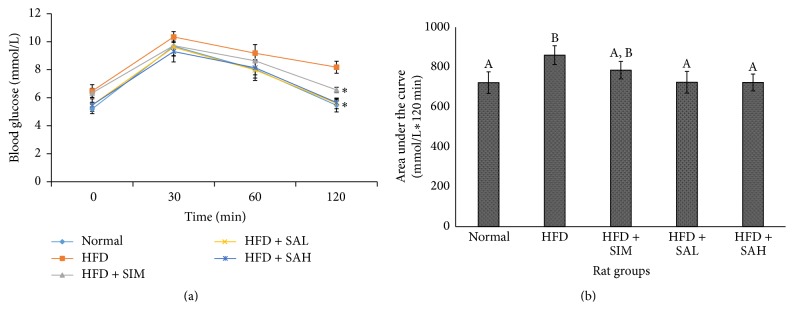
Effects of SA on oral glucose tolerance test in HFD-fed rats. (a) Glycemic response to 2 g glucose/kg BW over 120 min. (b) Area under the curve for glucose after 120 min. Groups are the same as Table 1. ^*∗*^ indicates statistically significant difference (*P* < 0.05) in comparison with HFD. Different letters on bars in (b) indicate a statistically significant difference (*P* < 0.05).

The OGTT, insulin, HOMA-IR, and lipid profile results are shown in Table 3 and Figure 2. The results indicated that SA improved lipid profile values and insulin sensitivity (HOMA-IR) of rats, although only the triglycerides were significantly different (*P* < 0.05) in comparison with the HFD group (Table 3). Moreover, OGTT results showed that the HFD + SAH and HFD + SAL groups, unlike the HFD and HFD + SIM groups, had better insulin sensitivity (Figure 2); the HFD + SAH and HFD + SAL groups had better glycemic response on OGTT and lower area under the curve for glucose over 120 min of OGTT (*P* < 0.05).

### 3.3. Serum Adiponectin and Leptin

Table 3 shows the serum adiponectin and leptin results. From the table, elevated leptin and reduced adiponectin levels were observed. The leptin level was significantly lower in the HFD + SIM, HFD + SAH, and HFD + SAL groups compared to the HFD group (*P* < 0.05). Adiponectin level in the HFD + SIM group was also lower than that of the HFD group, while the HFD + SAH and HFD + SAL groups had higher levels, despite being not significantly different from the HFD group.

### 3.4. Hepatic and Adipose Tissue mRNA Levels of Insulin Signaling Genes

**Figure 3:**
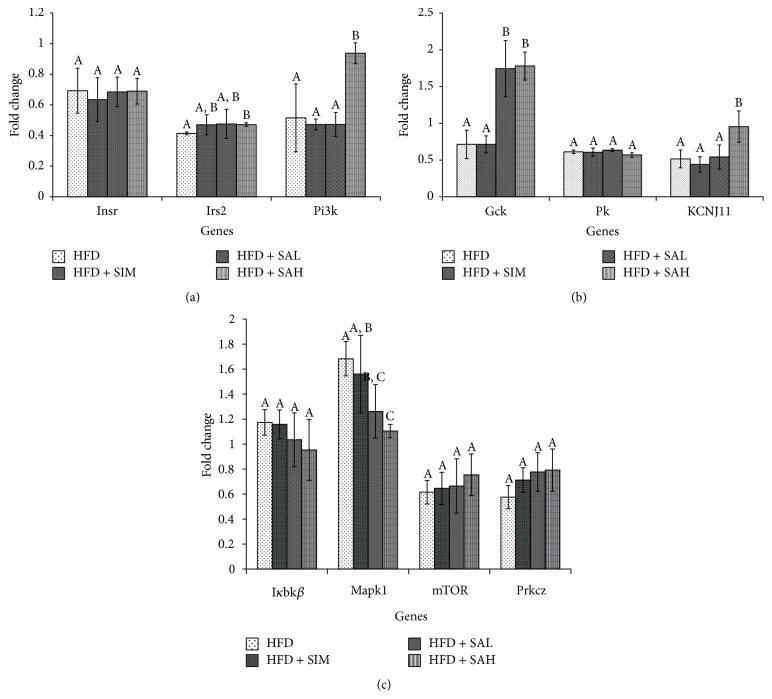
Effects of SA on hepatic mRNA levels of (a) insulin receptor (Insr), insulin receptor substrate (Irs) 2, and phosphoinositide-3-kinase (PI3K), (b) glucokinase (Gck), potassium inwardly rectifying channel, subfamily J, member 11 (KCNJ11), and pyruvate kinase-liver isoform (L-Pk), and (c) mammalian target of rapamycin (mTOR), protein kinase C, zeta (Prkcz), inhibitor of kappa light polypeptide gene enhancer in B-cells, kinase beta (I*κ*BK*β*), and mitogen-activated protein kinase (MAPK) 1 in HFD-fed rats. Groups are the same as Table 1. Different letters on bars representing each group indicate a statistically significant difference (*P* < 0.05).

**Figure 4:**
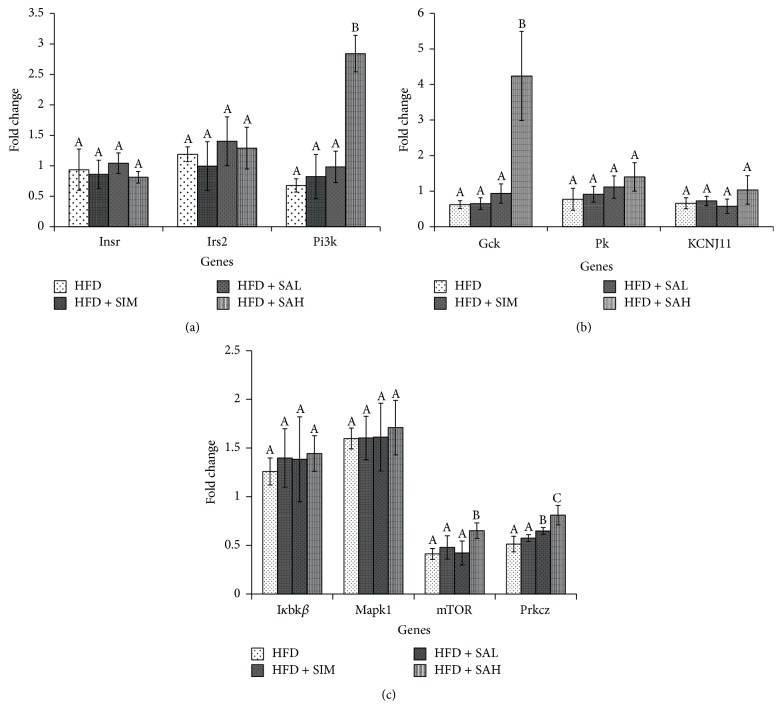
Effects of SA on adipose tissue mRNA levels of (a) insulin receptor (Insr), insulin receptor substrate (Irs) 2, and phosphoinositide-3-kinase (PI3K), (b) glucokinase (Gck), potassium inwardly rectifying channel, subfamily J, member 11 (KCNJ11), and pyruvate kinase-liver isoform (L-Pk), and (c) mammalian target of rapamycin (mTOR), protein kinase C, zeta (Prkcz), inhibitor of kappa light polypeptide gene enhancer in B-cells, kinase beta (I*κ*BK*β*), and mitogen-activated protein kinase (MAPK) 1 in HFD-fed rats. Groups are the same as Table 1. Different letters on bars representing each group indicate a statistically significant difference (*P* < 0.05).

Figures 3 and 4 show the effects of SA on hepatic and adipose tissue mRNA levels of insulin signaling genes, respectively. The HFD + SAH group had upregulation of the glucokinase (Gck), potassium inwardly rectifying channel, subfamily J, member 11 (KCNJ11), mammalian target of rapamycin (mTOR), phosphoinositide-3-kinase (Pi3k), and prkcz-protein kinase C, zeta (Prckz) genes in both liver and adipose tissues. In addition, it had downregulation of the inhibitor of kappa light polypeptide gene enhancer in B-cells, kinase beta (I*κ*bk*β*), and mitogen-activated protein kinase (Mapk1) in the liver, while it upregulated pyruvate kinase (Pk) in the adipose tissue. Other genes involved in the insulin signaling pathway were not changed by SA (Figures 3 and 4).

## 4. Discussion

As can be recalled, the HFD + SIM, HFD + SAH, and HFD + SAL groups had lower weight gains in comparison with the HFD group. Simvastatin has weight-reducing properties [12] and hence the lower weight gain observed. SA, on the other hand, has not been reported to reduce weight, and the observations from this study suggest that it may regulate weight gain. Therefore, we hypothesized that this effect may have been due to differential sialylation or resialylation of glycoprotein or glycolipids with implications on weight gain. Moreover, Zinc *α*2-glycoprotein (ZAG), a glycoprotein, is an established marker for fat catabolism [13], and dietary SA may have affected its sialylation or that of similar yet unknown glycoprotein adipokines, with resultant changes in their functions due to changes in sialylation status. This is an area worth evaluating further, especially since the metabolism of such glycoproteins may in itself be influenced by the degree of sialylation [14, 15]. Additionally, the changes in lipid profiles and insulin sensitivity markers in the HFD + SAH and HFD + SAL groups may also have been due to differential sialylation of glycoproteins like ZAG, with resultant modulation of various metabolic pathways. The lower weight gains in the HFD + SAH and HFD + SAL groups may also have contributed to the metabolic outcomes observed in these groups, since lower weights have been associated with lower lipid profiles and improved insulin sensitivity [8]. Elevated leptin and reduced adiponectin levels are associated with cardiometabolic diseases [16], and in the present study, a similar pattern was observed for the HFD group (Table 3). Conversely, SA reduced leptin level significantly lower than that in the HFD group and increased the adiponectin level, albeit not significantly. Simvastatin, however, reduced both the leptin and adiponectin levels. The effects of SA on adiponectin and leptin indicated that it could improve cardiometabolic indices, since these markers are predictors of metabolic diseases [16].

The gene expression data from this study showed that the HFD + SIM and HFD groups had transcriptional changes of insulin signaling genes that tended towards insulin resistance. Furthermore, the data showed that the HFD + SAH group attenuated the HFD-induced transcriptional changes, which tended towards improved insulin signaling. Specifically, it upregulated the expression of a central mediator of the intracellular signal transduction of insulin sensing (Pi3k), whose transcriptional downregulation has been linked with obesity-induced insulin resistance [17, 18]. Similarly, the upregulation of mTOR [19] and prkcz [20] and downregulation of Mapk1 [21] and I*κ*bk*β* [22] especially in the HFD + SAH group suggested improved insulin sensitivity, which may have been mediated through increased dephosphorylation of insulin receptor substrate (IRS) with consequent increase in IRS-mediated insulin action via activation of Pi3k [17, 23]. The data in this study support this hypothesis, since the expression of Pi3k was increased but not that of IRS. Also, in the present study, SA upregulated Gck (liver and adipose tissues) and Pk (adipose tissue), which are associated with enhanced glucose sensing and homeostasis and elevated cellular adenosine triphosphate (ATP) levels [24]. Elevated cellular ATP will consequently increase KCNJ11, which is reported to regulate ion channels involved in glucose sensing [25].

**Figure 5:**
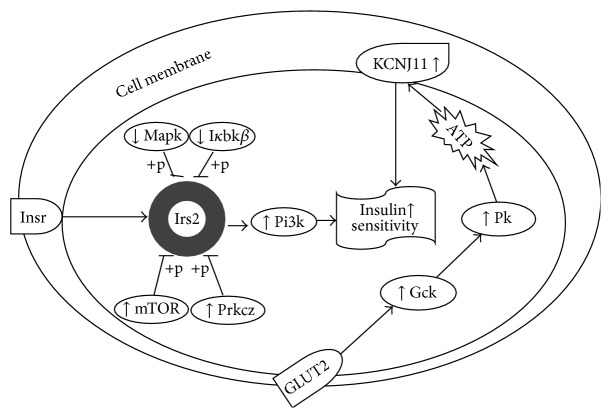
Proposed schematic showing targets of SA transcriptional regulation of insulin signaling pathway. SA prevents insulin resistance in HFD-fed rats by influencing the transcriptional regulation of multiple genes. Gck: glucokinase; I*κ*bk*β*: inhibitor of kappa light polypeptide gene enhancer in B-cells, kinase beta; IRS: insulin receptor substrate; KCNJ11: potassium inwardly rectifying channel, subfamily J, member 11; Mapk: mitogen-activated protein kinase; mTOR: mammalian target of rapamycin; Pi3k: phosphoinositide-3-kinase; Pk: pyruvate kinase; and Prkcz: protein kinase C, zeta.

Taken together, the data showed that although simvastatin improved lipid profiles, it increased insulin resistance, as reported previously [26]. SA, on the other hand, improved lipid profiles and prevented HFD-induced insulin resistance. Serum insulin levels in the HFD + SIM, HFD + SAH, and HFD + SAL groups were similar, but insulin sensitivity was better in the HFD + SAH and HFD + SAL groups, suggesting that the effects of SA may have been mediated at the insulin signaling level. The effects of SA on mRNA levels of hepatic and adipose tissue insulin signaling genes confirmed our hypothesis. However, despite improved insulin sensitivity in the HFD + SAH and HFD + SAL groups, only the HFD + SAH group showed upregulation of the insulin signaling genes, suggesting that the effects of SA may be both transcriptional (at higher doses) and nontranscriptional (at lower and higher doses). Earlier, we hypothesized that the improvements in weight and other metabolic indices observed due to SA could have been due to its effects on glycoprotein sialylation. Moreover, glycoprotein sialylation is reportedly influenced by the degree of metabolic flux [14, 15], and dietary SA administration could have influenced the flux towards increased sialylation of glycoprotein structures that influenced cardiometabolic indices. These effects induced by SA are likely to be transient, since reduced SA flux could rapidly reverse any changes produced. However, transcriptionally mediated changes at higher doses of SA may produce longer-lasting effects [26, 27]. Therefore, from the findings in this study, we propose that SA may be able to prevent insulin resistance through transcriptional regulation of insulin signaling genes (Figure 5) and nontranscriptional mechanisms, depending on the concentration used.

## 5. Conclusions

We demonstrated that SA prevents HFD-induced insulin resistance through transcriptional and nontranscriptional mechanisms. At lower doses, sialylation of glycoprotein targets may be responsible for the preventive effects of SA against insulin resistance. In addition, at higher doses, transcriptional regulation of insulin signaling genes may provide longer-lasting effects. These findings are worth evaluating further.

## Figures and Tables

**Table 1:** Food composition and animal groups.

Animal group	Normal pellet (%)	Cholesterol/cholic acid (%)	Palm oil (%)	Starch (%)	Others
Normal	100				
HFD	65	5	20	10	
HFD + SIM	65	5	20	10	Simvastatin (10 mg/kg BW/day)
HFD + SAL	65	5	20	10	50 mg/kg BW/day sialic acid
HFD + SAH	65	5	20	10	400 mg/kg BW/day sialic acid

Normal pellet was acquired commercially while other diets were made in-house. HFD: high fat diet, SIM: simvastatin, SAL: low dose sialic acid, and SAH: high dose sialic acid.

**Table 2:** Names, accession number, and primer sequences used in the study.

	Accession number	Left sequence	Right sequence
Irs2	NM_001168633	AGGTGACACTATAGAATAAGGCACTGGAGCCTTAC	GTACGACTCACTATAGGGAGCAGCACTTTACTCTTTCAC
Kcnj11	NM_031358	AGGTGACACTATAGAATACTACTTCAGGCAAAACTCTG	GTACGACTCACTATAGGGAGAACTTTCCAATATTTCTTTT
Insr	NM_017071	AGGTGACACTATAGAATAAGCTGGAGGAGTCTTCAT	GTACGACTCACTATAGGGAAAGGGATCTTCGCTTT
Gck	NM_001270849	AGGTGACACTATAGAATAATCTTTTGCAACACTCAGC	GTACGACTCACTATAGGGATTGTTGGTGCCCAGA
Pk	NM_012624	AGGTGACACTATAGAATATCGGAGGTGGAAATTG	GTACGACTCACTATAGGGACTCTGGGCCGATTTT
B2m^*∗*^	NM_012512	AGGTGACACTATAGAATAATGCTTGCAGAGTTAAACA	GTACGACTCACTATAGGGATGCATAAAATATTTAAGGTAAGA
Hprt1^*∗*,#^	NM_012583	AGGTGACACTATAGAATATCCTCATGGACTGATTATG	GTACGACTCACTATAGGGACTGGTCATTACAGTAGCTCTT
Mapk1	NM_053842	AGGTGACACTATAGAATACATTTTTGAAGAGACTGCTC	GTACGACTCACTATAGGGAAACTCTCTGGACTGAAGAAT
Prkcz	NM_022507	AGGTGACACTATAGAATACTTTAACAGGAGAGCGTACT	GTACGACTCACTATAGGGATATTGTCATGTTTCCGAGAT
I*κ*bk*β*	NM_053355	AGGTGACACTATAGAATACTTGAACTTAAAGCTGGTTC	GTACGACTCACTATAGGGAACATTTTACTGTTGTCAAAGAG
KanR^*∗∗*^			
Mtor	NM_019906	AGGTGACACTATAGAATATGGAACTTCGAGAGATGAG	GTACGACTCACTATAGGGATCACTTCAAACTCCACATAC
Actb^*∗*^	NM_031144	AGGTGACACTATAGAATAAACTACATTCAATTCCATCA	GTACGACTCACTATAGGGATAAAACGCAGCTCAGTAAC
Pik3r1	NM_013005	AGGTGACACTATAGAATACATCAGTATTGGCTTACG	GTACGACTCACTATAGGGATCATTTACTTCTTCCCTTGA

^*∗*^Housekeeping genes. ^#^Normalization gene. Underlined sequences are left and right universal left and right sequences (tags). ^*∗∗*^Internal control supplied by Beckman Coulter, Inc. (Miami, FL, USA), as part of the GeXP kit. RT conditions were 48°C for 1 min, 37°C for 5 min, 42°C for 60 min, and 95°C for 5 min and then hold at 4°C. PCR conditions were initial denaturation at 95°C for 10 min, followed by two-step cycles of 94°C for 30 sec and 55°C for 30 sec, ending in a single extension cycle of 68°C for 1 min. Actb: beta actin; B2m: beta-2 microglobulin; Hprt1: hypoxanthine phosphoribosyltransferase 1; Irs2: insulin receptor substrate 2; Kcnj11: potassium inwardly rectifying channel, subfamily J, member 11; Insr: insulin receptor; Gck: glucokinase; KanR: kanamycin resistance; Mapk1: mitogen-activated protein kinase 1; Pk: pyruvate kinase; Prkcz: protein kinase C, zeta; mTOR: mammalian target of rapamycin (serine/threonine kinase); I*κ*bk*β*: inhibitor of kappa light polypeptide gene enhancer in B-cells, kinase beta; Pik3r1: phosphoinositide-3-kinase, regulatory subunit 1 (alpha).

**Table 3:** Final body weights, food intake, and biochemical parameters.

Rat groups	Final body weight (g)	Food intake (Kcal/kg BW/day)	Chol. (mmol/L)	Trig. (mmol/L)	LDL (mmol/L)	HDL (mmol/L)	LDL/HDL	Insulin (pg/mL)	HOMA-IR	Leptin (ng/mL)	Adiponectin (mg/mL)
Normal	384 ± 22.9^a^	215.54 ± 33.5^a^	1.55 ± 0.43^a^	0.62 ± 0.15^a^	0.28 ± 0.11^a^	1.18 ± 0.35^a^	0.24 ± 0.04^a^	495 ± 51.3^a^	1.91 ± 0.23^a^	1.17 ± 0.39^a,b,c^	36.45 ± 0.35^a^
HFD	395.2 ± 16.8^a^	215.04 ± 37.45^a^	7.47 ± 1.13^b^	1.21 ± 0.38^b^	4.98 ± 1.03^b^	1.05 ± 0.13^a^	4.77 ± 0.98^b^	513.3 ± 38.8^a^	2.46 ± 0.22^b^	1.53 ± 0.24^a^	30.89 ± 3.40^b^
HFD + SIM	375.7 ± 53.4^a^	215.67 ± 36.60^a^	4.99 ± 1.11^c^	0.63 ± 0.18^a^	3.6 ± 1.1^b^	1.04 ± 0.17^a^	3.46 ± 0.94^b^	602.1 ± 145.7^a^	2.83 ± 0.79^a,b^	0.76 ± 0.09^b^	26.66 ± 0.18^c^
HFD + SAL	379.3 ± 41.3^a^	216.16 ± 37.27^a^	5.68 ± 2.18^b,c^	0.54 ± 0.07^a^	4.48 ± 1.81^b^	1.04 ± 0.28^a^	4.14 ± 0.79^b^	521.25 ± 118.65^a^	2.12 ± 0.56^a,b^	1.04 ± 0.16^c^	34.81 ± 0.92^b^
HFD + SAH	368.5 ± 14.6^a^	215.53 ± 36.38^a^	5.05 ± 2.07^b,c^	0.54 ± 0.07^a^	3.67 ± 1.58^b^	1.08 ± 0.27^a^	3.31 ± 1.08^b^	512.77 ± 90.3^a^	2.08 ± 0.42^a,b^	1.02 ± 0.20^b,c^	40.52 ± 5.69^a,b^

Data represent mean ± SD (*n* = 6). Different alphabet in each column denotes significant difference (*P* < 0.05) in Tukey's multiple comparison test. Groups are the same as Table 1. HDL: high-density lipoprotein; HOMA-IR: homeostatic model assessment of insulin resistance; LDL: low-density lipoprotein; Chol: cholesterol; SIM: simvastatin; Trig: triacylglycerol; HFD: high fat diet; SAL: low dose sialic acid; SAH: high dose sialic acid.

## Abbreviations

Gck: Glucokinase
HFD: High fat diet
HOMA-IR: Homeostatic model assessment of insulin resistance
I*κ*bk*β*: Inhibitor of kappa light polypeptide gene enhancer in B-cells, kinase beta
IRS: Insulin receptor substrate
KCNJ11: Potassium inwardly rectifying channel, subfamily J, member 11
Mapk: Mitogen-activated protein kinase
mTOR: Mammalian target of rapamycin
OGTT: Oral glucose tolerance test
Pi3k: Phosphoinositide-3-kinase
Pk: Pyruvate kinase
Prkcz: Protein kinase C, zeta
SA: Sialic acid
SAH: High-dose sialic acid
SAL: Low dose sialic acid.
